# Death of Dr. John M. Johnson

**Published:** 1886-06

**Authors:** 


### DEATH OF DR. JOHN M. JOHNSON.

The medical profession of Atlanta has lost its oldest, most popular and best-beloved member. Dr. John M. Johnson died on the 18th of May of a complication of disorders from which he had long suffered. He was born in 1810, and was consequently in the seventy-sixth year of his age. He was born and reared in the western part of Kentucky, and practiced his profession there with diligence and success for many years, ranking, as he would have done anywhere, among the most eminent of his calling. The stirring political events of the period aroused his patriotism and demanded his participation. He held many positions of public trust, and at the breaking out of the war between the States was a member of the State Senate. His prominence and activity on the Southern side caused the issue of an order for his arrest by the Federal authorities. He narrowly escaped, and joined the Southern forces under General Albert Sidney Johnson. From that time his life and fortunes were with the South. After arduous services as a medical officer in the army, he established his residence in this city. Here he lived—honored, trusted, beloved, as few men have ever been—until the final summons reached him.

In this brief notice it is impossible to speak fitly of the powers of his intellect and the charm of his manner, which gave him eminence and reverence as a physician; of his expansive benevolence, as universal as the dews of heaven; of the wealth of music and poetry with which nature had endowed him; of the exhaustless and refining wit, whose scintillations delighted all who were favored with his instructive association. Our humble task is but to chronicle his death and place a simple chaplet upon his grave.

